# Supplementary material for: Pharmacokinetic-pharmacodynamic modeling of benznidazole and its antitrypanosomal activity in a murine model of chronic Chagas disease
Source: PLoS Negl Trop Dis. 2025 May 13;19(5):e0012968. doi: 10.1371/journal.pntd.0012968 (PMC12074391; doi:10.1371/journal.pntd.0012968)
Supplement: S1 Text — (DOCX) [file pntd.0012968.s001.docx]

**S1 Text. Determination of in vitro antitrypanosomal activity**

In vitro activity of benznidazole against *T. cruzi* amastigotes (Tulahuen WT strain) in 3T3 host cells was determined using a previously published high-content, image-based analysis [1].

The IC_90_ value for benznidazole in the assay medium (RPMI medium supplemented with 10% fetal calf serum) was determined to be ${IC}_{90,medium}$ = 17.4 µM. Since the binding of benznidazole in RPMI medium was minimal (<10%), an unbound fraction of $f_{unbound,medium}$  = 0.95 was assumed for scaling ${IC}_{90,medium}$ to ${IC}_{90,unbound}$, as follows:

${IC}_{90,unbound}= {IC}_{90,medium}\times f_{unbound,medium}$ . (1)

Here, ${IC}_{90,unbound}$ represents the unbound benznidazole concentration needed to achieve a 90% reduction in *T. cruzi* amastigote infection in 3T3 host cells. Benznidazole also exhibited low protein binding in mice plasma (32.3% bound). Accordingly, an unbound fraction of $f_{unbound,plasma}$ = 0.677 in mice plasma was used to correct for protein binding, as follows:

${IC}_{90,plasma}= \frac{{IC}_{90,unbound}}{f_{unbound,plasma}}$ . (2)

The derived total concentration of benznidazole in mice plasma ( ${IC}_{90,plasma}$= 24.7 µM, i.e. 6.43 µg/mL) was set as the target concentration in mice.

The protein binding of benznidazole in RPMI media and mouse plasma (male Swiss mice) was determined by ultracentrifugation at 37°C and was reported previously [2].

**Table A.** Protein binding and in vitro antitrypanosomal activity of benznidazole (in-house).

| **Parameter** | **Value** |
| --- | --- |
| In vitro ${IC}_{90,unbound}$ (µM) | 16.7^a^ (=4.35 µg/mL) |
| **In vitro** $\boldsymbol{IC}_{\boldsymbol{90}\boldsymbol{,}\boldsymbol{plasma}}$ **(µM)** | **24.7^b^ (=6.43 µg/mL)** |
| Protein binding in cell culture media (RPMI) (%) | <10^c^ |
| Protein binding in plasma, mice (%) | 32.3^d^ |

^a^Estimated from in vitro IC_90,medium_ (17.4 µM) in RPMI media (containing 10% FCS), and assuming 5% binding to assay media.

^b^IC_90_ against *T.cruzi* amastigotes, scaled to represent the total plasma concentration in mice needed to achieve 90% of maximum effect

^c^Binding to RPMI media measured at 50 ng/mL and 200 ng/mL benznidazole

^d^Binding to mouse plasma determined at 1000 ng/mL benznidazole

**References**

1. Sykes ML, Avery VM. Development and application of a sensitive, phenotypic, high-throughput image-based assay to identify compound activity against Trypanosoma cruzi amastigotes. International journal for parasitology Drugs and drug resistance. 2015;5(3):215-28.

2. Francisco AF, Jayawardhana S, Lewis MD, White KL, Shackleford DM, Chen G, et al. Nitroheterocyclic drugs cure experimental Trypanosoma cruzi infections more effectively in the chronic stage than in the acute stage. Scientific reports. 2016;6:35351.
