# Supplementary material for: Pharmacokinetic-pharmacodynamic modeling of benznidazole and its antitrypanosomal activity in a murine model of chronic Chagas disease
Source: PLoS Negl Trop Dis. 2025 May 13;19(5):e0012968. doi: 10.1371/journal.pntd.0012968 (PMC12074391; doi:10.1371/journal.pntd.0012968)
Supplement: S2 Text — (DOCX) [file pntd.0012968.s002.docx]

**S2 Text. Classification performance metrics**


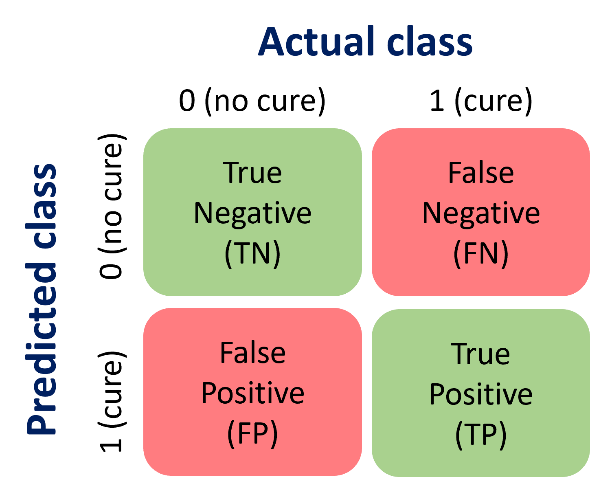


**Accuracy** is defined as the ratio of correctly classified mice to the total number of mice:

$$Accuracy=\frac{TN+TP}{TN+FP+FN+TP}$$

**Sensitivity (=Recall or True positive rate)** is defined as the ratio of mice correctly classified as cured over the total number of cured mice in reality.

$$Sensitivity=\frac{TP}{TP+FN}$$

**Specificity (True negative rate)** is defined as the ratio of mice correctly classified as not cured over the total number of not cured mice in reality.

$$Specificity=\frac{TN}{TN+FP}$$

**Precision (=positive predictive value)** is defined as the ratio of mice correctly classified as cured to the total number of mice classified as cured.

$$Precision=\frac{TP}{FP+TP}$$

**F1-score** considers both precision and recall

$$F1 score=\frac{2*(Recall*Precision)}{(Recall+Precision)}$$

**Matthew Correlation Coefficient** measures the correlation of the true classes *c* with the predicted labels *l* [1]:

$$MCC=\frac{{Cov}_{(c,l)}}{\sigma_{c}*\sigma_{l}}=\frac{TP*TN-FP*FN}{\sqrt{(TP+FP)(TP+FN)(TN+FP)(TN+FN)}}$$

Where ${Cov}_{(c,l)}$is the covariance of the true classes c and predicted labels l; σ_c_ and σ_l_ are the standard deviations, respectively. It is also referred to as the Pearson product-moment correlation coefficient between actual and predicted values [2]. MCC produces a high score (ranging from -1 worst; to +1 best) when the classifier correctly predicts most of the four confusion matrix categories (true positives, false negatives, true negatives, and false positives) [2]. This metric is considered more reliable for imbalanced datasets compared to accuracy and F1-score.

**References**

1. Chicco D, Tötsch N, Jurman G. The Matthews correlation coefficient (MCC) is more reliable than balanced accuracy, bookmaker informedness, and markedness in two-class confusion matrix evaluation. BioData mining. 2021;14(1):13.

2. Chicco D, Jurman G. The advantages of the Matthews correlation coefficient (MCC) over F1 score and accuracy in binary classification evaluation. BMC genomics. 2020;21(1):6.
