# Supplementary material for: Pharmacokinetic-pharmacodynamic modeling of benznidazole and its antitrypanosomal activity in a murine model of chronic Chagas disease
Source: PLoS Negl Trop Dis. 2025 May 13;19(5):e0012968. doi: 10.1371/journal.pntd.0012968 (PMC12074391; doi:10.1371/journal.pntd.0012968)
Supplement: S3 Text — (DOCX) [file pntd.0012968.s003.docx]

**S3 Text. Multivariate analysis**

***S3.1 Multivariate analysis - Methods***

Partial least squares – discriminant analysis (PLS-DA) was employed to investigate the relationship between a combination of PK/PD index parameters and parasitological cure. The analysis was conducted in R, utilizing the R package 'mdatools' [1].

Parasitological cure in *T. cruzi* infected mice was used as the binary outcome, with 'cure' coded as 1 and 'no cure' as -1. Prior to analysis, the PK/PD index parameters were scaled and centered. The model building process started with six predictor (x)-variables, namely C_MAX_, AUC_12_, AUC_24_, AUC_∞_, T>IC_90_, and duration of dosing. Internal cross-validation was performed using k-fold cross validation (k=7). The number of principal components was determined by considering explained variance (r^2^, calibration) and predicted variance (q^2^, prediction of the omitted group in cross validation) [2], as well as classification metrics. Redundant variables, identified as groups in the loading plot of the first two principal components, were omitted if their influence on r^2^ and q^2^ was judged negligible or redundant. Less important variables were eliminated based on their variable influence on projection (VIP). The classification performance of the final multivariate PLS-DA model was evaluated in comparison to univariate PLS-DA models, using optimized classification thresholds.

***S3.2 Multivariate analysis - Results***

The selection of the most relevant predictor variables from the complete set of PK/PD index parameters was guided by the VIP parameter (**Figure A**). In the final PLS-DA model, the following factors were identified as the most relevant predictors for class discrimination, ranked by decreasing importance: AUC_∞_, T>IC_90_ , and C_MAX_  (VIP scores: 1.47, 1.40 and 1.37). The regression coefficients, after scaling and centering, exhibited a consistent trend: AUC_∞_ had the highest absolute value (0.25, 95 % CI [0.20,0.31]), followed closely by T>IC_90_ (0.24, 95 % CI [0.18,0.30]) and C_MAX_ (0.23, 95 % CI [0.14,0.32]), with overlapping 95 % CIs. All variables had positive coefficients, indicating their contribution to an increased likelihood of cure according to the model's predictions.


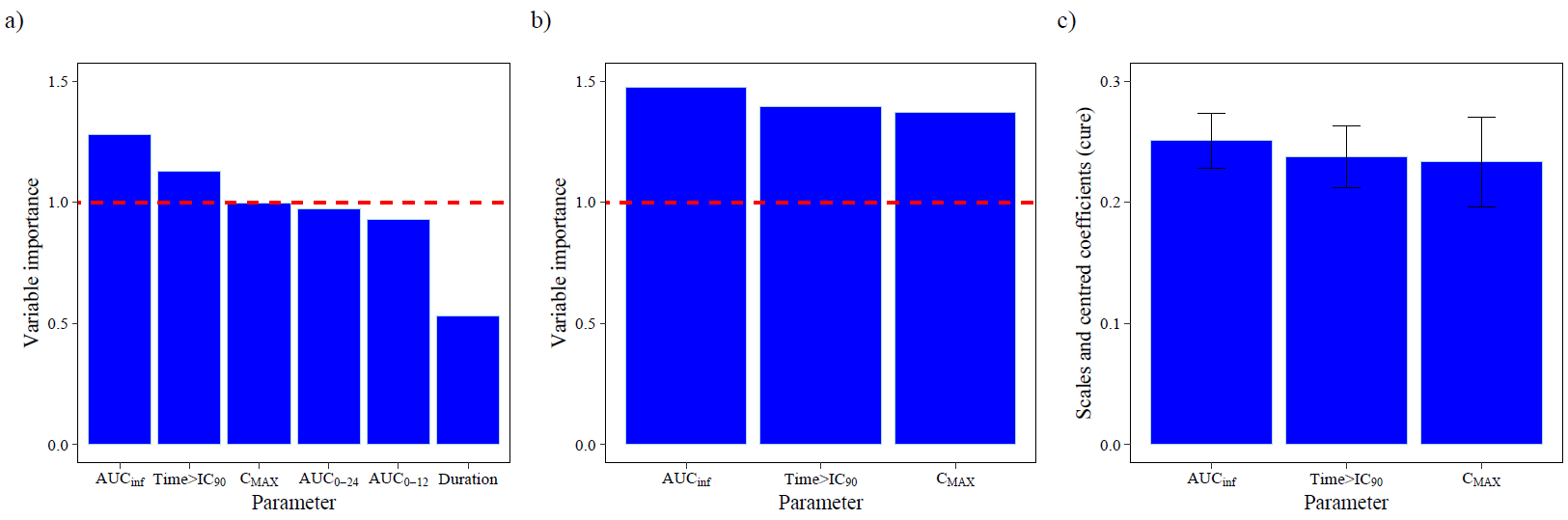


**Fig A.** Variable importance parameters for **a)** the preliminary PLS-DA model based on 4 latent variables and the complete set of PK/PD index parameters and **b)** the final PLS-DA model based on 1 latent variable and the subset of selected PK/PD index parameters. c) Scaled and centred regression coefficients for the final PLS-DA model; error bars indicate standard errors.

The scores and scores and loading plots of the first two latent variables of the final PLS-DA model are shown in **Figure B**.

**
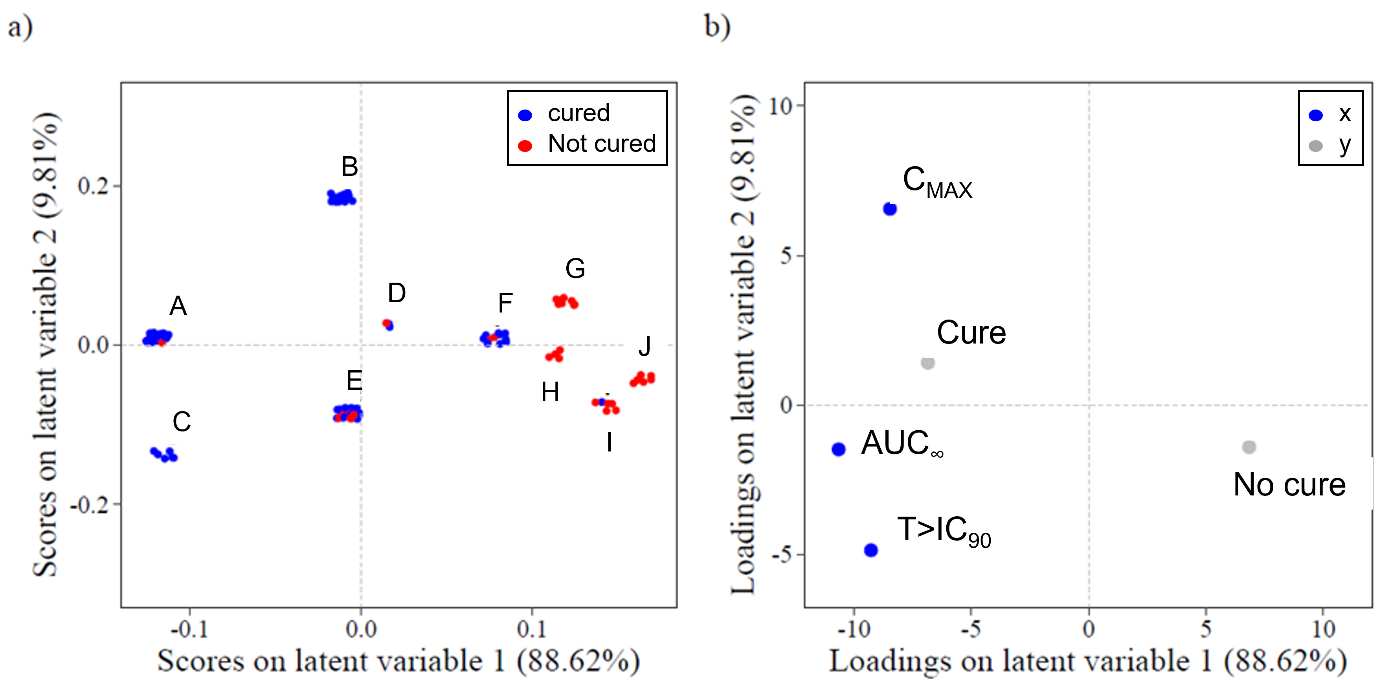
**

**Fig B.** **a)** Scores and **b)** loadings (x- and y- loadings) of the first two latent variables retained in the PLS-DA model. Explained x- variances are provided in brackets. Scores are shown for mice treated with different dosing regimens: A) 100 mg/kg, once daily, for 10 days, (B) 100 mg/kg, once daily, for 5 days, (C) 50 mg, twice daily, for 10 days, (D) 50 mg/kg, once daily, for 10 days, (E) 30 mg/kg, once daily, for 20 days, (F) 30 mg/kg, once daily, for 10 days, (G) 30 mg/kg, once daily, for 5 days, (H) 20 mg/kg, once daily, for 10 days, (I) 10 mg/kg, once daily, for 20 days, and (J) 10 mg/kg, once daily, for 10 days (see Table 1, main manuscript).

The largest part of the x-variance (88.6%) and y-variance (40%) was accounted for by the first latent variable, whereas the second and third latent variables only contributed to less than 10% of explained x-variance (**Figure C**).


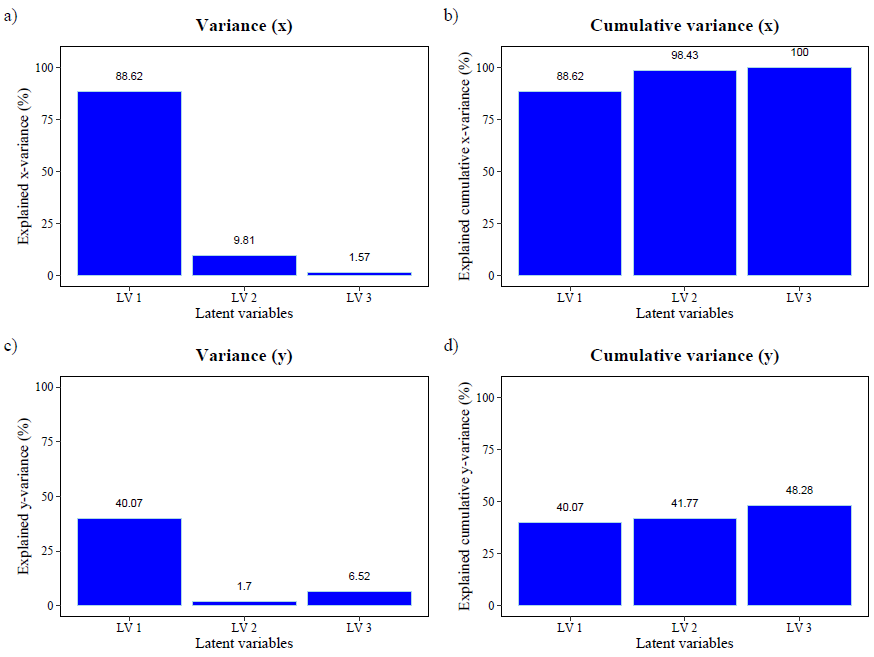


**Fig C.** Explained x- and y variances as a function of the number of latent variables (LVs) for the PLS-DA model based on a combination of AUC_∞_, T>IC_90_, C_MAX_.

Similar classification performances were observed for PLS-DA models constructed using one, two, or three latent variables (**Figure D** and **Table A**). Therefore, the least complex PLS-DA model, which relies on one latent variable, was chosen for further evaluation of univariate vs multivariate model performances.


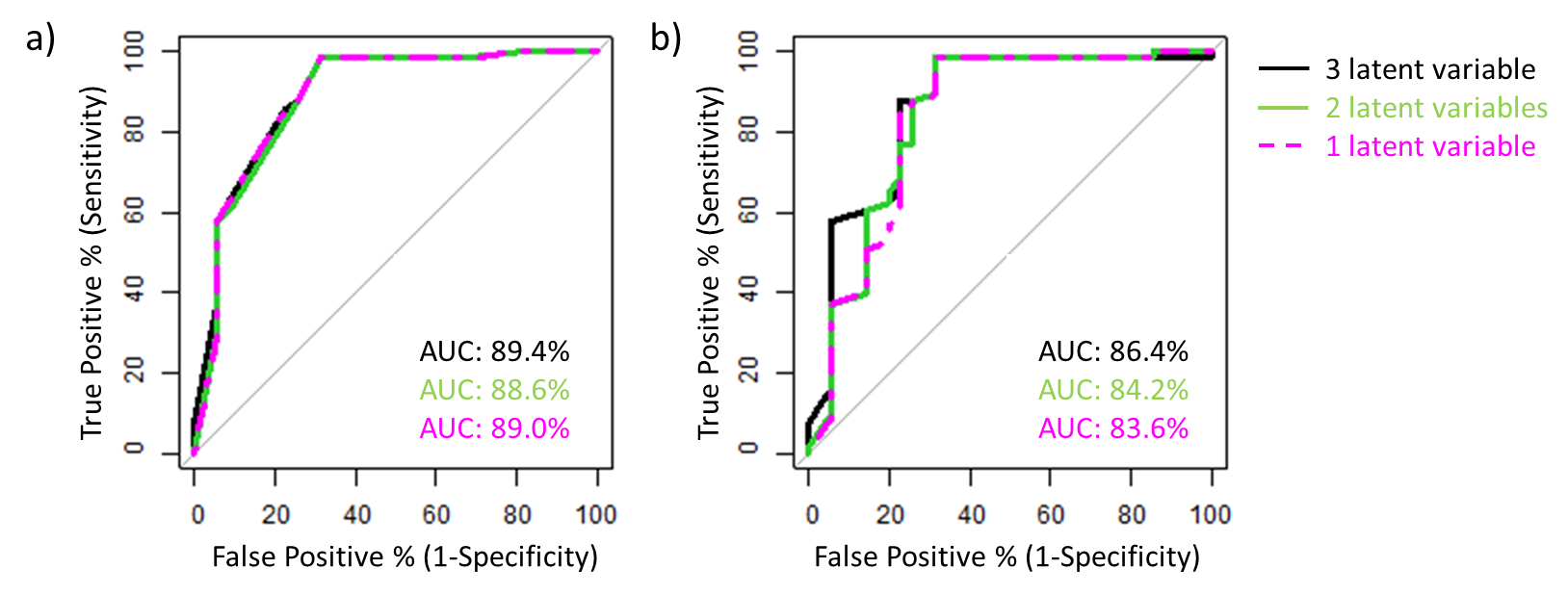


**Fig D.** Receiver Operating Characteristics (ROC) curves for different numbers of latent variables included in the PLS-DA model, based on **a)** the calibration set (fitting) and **b)** cross validation (performed with 7 groups, divided with the venetian blind procedure).

**Table A.** Classification performance for multivariate PLS-DA regression models as a function of number of latent variables, based on default and optimized (in blue) accuracy thresholds.

| **Parameter** | **1 latent variable** | | **2 latent variables** | | **3 latent variables** | |
| --- | --- | --- | --- | --- | --- | --- |
| **Classification threshold** | Default:  0 | Optimal  -0.086 | Default:  0 | Optimal  -0.075 | Default:  0 | Optimal  0.014 |
| ***Calibration set*** |  |  |  |  |  |  |
| **Accuracy** | 0.84 | 0.9 | 0.84 | 0.9 | 0.90 | 0.9 |
| **Sensitivity** | 0.88 | 0.99 | 0.88 | 0.99 | 0.99 | 0.99 |
| **Specificity** | 0.74 | 0.69 | 0.74 | 0.69 | 0.69 | 0.69 |
| **F1 score** | 0.88 | 0.93 | 0.88 | 0.93 | 0.93 | 0.93 |
| **ROC (%)** | 89.0 (81.6 - 96.4) | | 88.6 (81.1 - 96.1) | | 89.4 (82.4 -96.4) | |
| ***Cross validation*** |  |  |  |  |  |  |
| **Accuracy** | 0.83 | 0.86 | 0.83 | 0.86 | 0.86 | 0.86 |
| **Sensitivity** | 0.88 | 0.93 | 0.89 | 0.93 | 0.93 | 0.93 |
| **Specificity** | 0.74 | 0.69 | 0.69 | 0.69 | 0.69 | 0.69 |
| **F1 score** | 0.88 | 0.90 | 0.88 | 0.90 | 0.90 | 0.90 |
| **ROC (%)** | 83.6 (74.0 - 93.4) | | 84.2 (74.82 - 93.7) | | 86.4 (78.0 - 94.8) | |

Abbreviations: ROC, area under the Receiver Operating Characteristics (ROC) curve.

**Fig E** compares the performance of the final multivariate PLS-DA model (which incorporates AUC_∞_, T>IC_90_, and C_MAX_) with univariate analysis. Importantly, there was no evidence of overfitting, as the differences in model performance between the calibration and internal cross-validation datasets were generally small. In terms of goodness-of-fit diagnostics, the final multivariate PLS-DA model exhibited slightly superior performance compared to univariate regression analysis, explaining 40 % of the y-variance, in contrast to 33.5 % (AUC_∞_) or less (**Figure E.**, panel a).

In terms of classification performance, there was no significant improvement between the multivariate PLS-DA model and univariate regression analysis with either AUC_∞_ or T>IC_90_. These models yielded identical values for accuracy, sensitivity, and specificity when optimized classification thresholds were applied (**Figure E.**, panel b-d). Corresponding ROC curves are shown in **Figure F**.

Univariate regression with C_MAX_ showed slightly lower accuracy, with differences less than 10% compared to AUC_∞_ or T>IC_90_ (calibration dataset). In terms of areas under the ROC curve, no significant differences were found between multivariate and univariate models, regardless of whether AUC_∞_, T>IC_90_, or C_MAX_ was applied. Additional performance metrics are detailed in **Table B**.


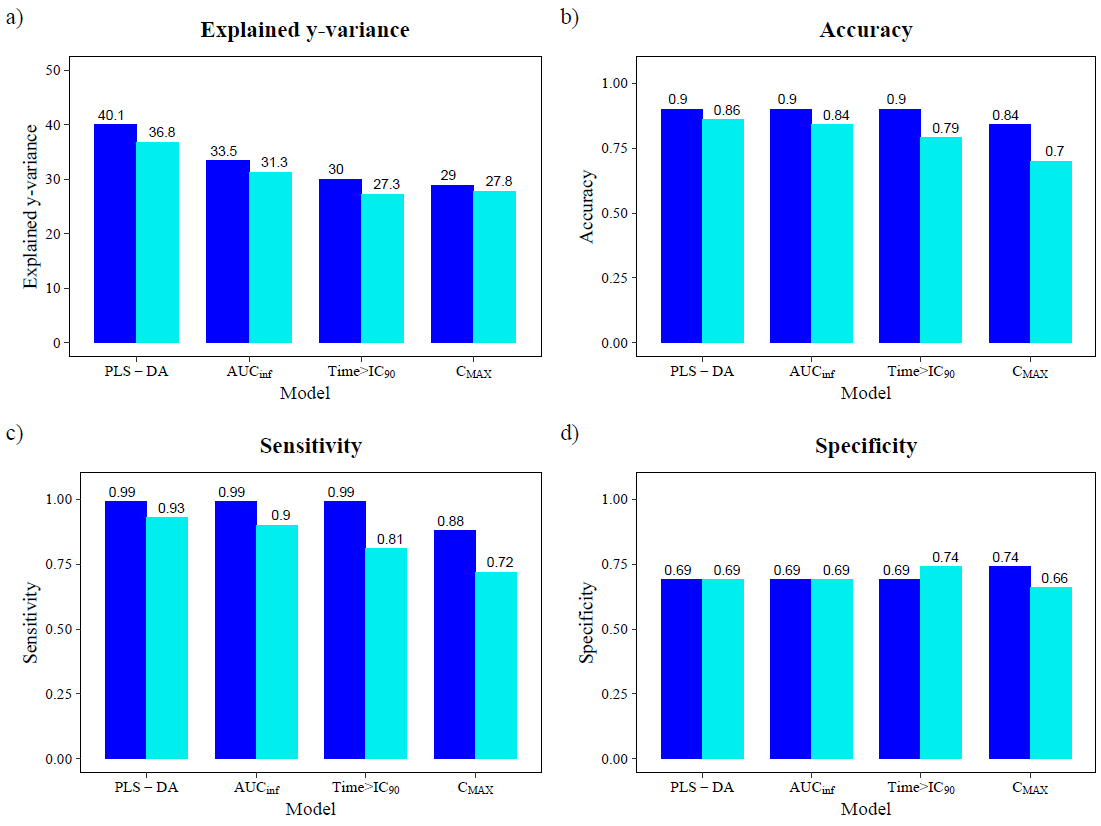


**Fig E.** Model performance for multivariate (AUC_∞_ + Time above ${IC}_{90,plasma}$ + C_MAX_, 1 latent variable) and univariate PLS-DA models in terms of a) explained y – variance, b) accuracy, c) sensitivity and d) specificity (based on optimized classification thresholds). Model performances are shown for the calibration (dark blue) and internal cross – validation (light blue) datasets.


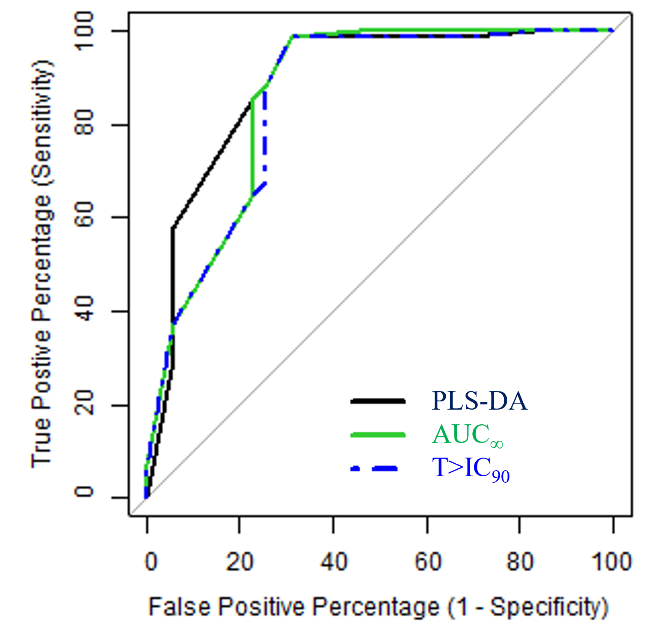


**Fig F.** Receiver Operating Characteristics (ROC) curves for the final multivariate PLS-DA model (AUC_∞_ + T>IC_90_ + C_MAX_) or univariate regression with either AUC_∞_ or T>IC_90_ as predictors of efficacy.

**Table B.** Classification performance for univariate and multivariate PLS-DA regression models, based on optimized accuracy thresholds.

|  | **Univariate regression** | | | **Multivariate regression (PLS-DA)** | | |
| --- | --- | --- | --- | --- | --- | --- |
| **Parameter** | **C_MAX_**  **(µg/mL)** | **AUC_∞_**  **(µg**×**h/mL)** | **Time above IC_90,plasma_ (days)** | **1 latent variable^a^** | **2 latent variables** | **3 latent variables** |
| ***Optimized threshold*** | 0.082 | -0.064 | 0.069 | **-0.086** | -0.075 | 0.014 |
| ***Calibration set*** |  |  |  |  |  |  |
| **Accuracy** | 0.84 | 0.90 | 0.90 | **0.90** | 0.90 | 0.90 |
| **Sensitivity** | 0.88 | 0.99 | 0.99 | **0.99** | 0.99 | 0.99 |
| **Specificity** | 0.74 | 0.69 | 0.69 | **0.69** | 0.69 | 0.69 |
| **F1 score** | 0.88 | 0.93 | 0.93 | **0.93** | 0.93 | 0.93 |
| **ROC (%)** | 87.0 | 86.4 | 85.3 | **89.0** | 88.6 | 89.4 |
| ***Cross validation*** |  |  |  |  |  |  |
| **Accuracy** | 0.70 | 0.84 | 0.79 | **0.86** | 0.86 | 0.86 |
| **Sensitivity** | 0.72 | 0.90 | 0.81 | **0.93** | 0.93 | 0.93 |
| **Specificity** | 0.74 | 0.69 | 0.69 | **0.69** | 0.69 | 0.69 |
| **F1 score** | 0.77 | 0.89 | 0.84 | **0.90** | 0.90 | 0.90 |
| **ROC (%)** | 77.5 | 83.5 | 80.0 | **83.6** | 84.2 | 86.4 |

Abbreviations: ROC, area under the Receiver Operating Characteristics (ROC) curve. ^a^Final PLS-DA model (in bold).

**References**

1. Kucheryavskiy S. mdatools – R package for chemometrics. Chemometrics and Intelligent Laboratory Systems. 2020;198.

2. Eriksson L, AB U. Multi- and Megavariate Data Analysis: Basic Principles and Applications. 1: Umetrics; 2006.
