# Supplementary material for: Pharmacokinetic-pharmacodynamic modeling of benznidazole and its antitrypanosomal activity in a murine model of chronic Chagas disease
Source: PLoS Negl Trop Dis. 2025 May 13;19(5):e0012968. doi: 10.1371/journal.pntd.0012968 (PMC12074391; doi:10.1371/journal.pntd.0012968)
Supplement: S4 Text — (DOCX) [file pntd.0012968.s004.docx]

**S4 Text. Simulation of benznidazole exposure in mice vs human**

The achievement of PK/PD targets in humans for the standard clinical dose (5 mg/kg/day, divided into two doses, for 56 days) was explored, based on the assumption that the mouse model has translational value for predicting benznidazole efficacy in humans. In addition, plasma-concentration time profiles in human for shorter treatment durations (5 mg/kg/day, divided into two doses, for 28 days) and lower daily doses (2.5 mg/kg/day, once daily, for 28 days), as recently been explored in the BENDITA study, were simulated [1].

Plasma concentration-time profiles for benznidazole in humans were simulated for an adult weighing 60 kg. Simulations were based on pharmacokinetic model parameters derived from a meta-analysis of benznidazole pharmacokinetics in humans, following a single 100 mg oral dose of benznidazole in adults) [2]. The parameters used in our simulations were as follows: CL/F = 2.04 L/h, V/F = 39.19 L/h, and K_A_ = 1.16 h^-1^.

Additionally, plasma concentration-time profiles in mice were simulated for 100 mg/kg benznidazole administered once daily for 10 days, corresponding to the reference experiment in mice associated with more than 90% cure of *T. cruzi* infected mice. Plasma exposure levels were compared between species, with the assumption that differences in plasma protein binding between mouse and human plasma were negligible [3, 4]. Simulations, based on population mean PK parameters estimates, were performed using Berkeley Madonna (v. 8.3.18). The graphical representation was accomplished using GraphPad Prism (v.9.3.1).

In **Figure A**, an overlay of simulated plasma concentration-time profiles for benznidazole in humans and in mice is presented. For the standard dosing regimen in human, simulated total plasma exposure was found to be 4.2-fold higher (AUC_∞_ = 8235 μg×h/mL) compared to mice (AUC_∞_ = 1950 μg×·h/mL). Similarly, the duration above the target concentration showed a 7.8-fold longer T>IC_90_ in humans (22.5 days) compared to mice (2.9 days). In contrast, C_MAX_ values were 6.2-fold higher in mice (46.1 µg/mL) than in humans (7.4 µg/mL).

It is important to note that the optimal dosing regimen of benznidazole in human is still a matter of debate, and shorter treatment durations as well as reduced daily doses are currently investigated [1, 5]. The total plasma exposure in human receiving benznidazole for only 28 days yielded a median AUC_∞_ of 4112 μg×h/mL (2.1-fold higher than in mice), and a 3.8-fold longer T>IC_90_ (10.9 days) with no impact on C_MAX_ compared to 56 days of dosing. Reduction of the daily benznidazole dose to 2.5 mg/kg for 28 days yielded a median AUC_∞_ of 2059 μg×h/mL (similar to AUC_∞_ in mice), but a median of zero days T>IC_90_ and even stronger species-discrepancies in C_MAX_. Validating the translational value of exposure levels in mice is challenging without a definite endpoint of cure in humans and the uncertainty regarding the sufficient exposure levels in human.

**Fig A.** Simulated mean plasma concentration-time profile for benznidazole in mice and in humans for the following dosing regimens: In mice, 100 mg/kg benznidazole, once daily for 10 days (reference experiment in mice, body weight 25 g, blue line). In human (for a 60 kg adult), 5 mg/kg/day, divided in two doses, for 56 days (standard clinical dose in human, light green line) or for 28 days of dosing (dark green line). In addition, the simulated plasma concentration-time profile for half the daily dose (2.5 mg/kg/day benznidazole for 28 days is shown, red line). The grey area indicates the generally accepted therapeutic range of benznidazole in plasma (3 to 6 µg/mL).

**References**

1. Torrico F, Gascón J, Barreira F, Blum B, Almeida IC, Alonso-Vega C, et al. New regimens of benznidazole monotherapy and in combination with fosravuconazole for treatment of Chagas disease (BENDITA): a phase 2, double-blind, randomised trial. The Lancet Infectious diseases. 2021;21(8):1129-40.

2. Wiens MO, Kanters S, Mills E, Peregrina Lucano AA, Gold S, Ayers D, et al. Systematic Review and Meta-analysis of the Pharmacokinetics of Benznidazole in the Treatment of Chagas Disease. Antimicrobial agents and chemotherapy. 2016;60(12):7035-42.

3. Workman P, White RA, Walton MI, Owen LN, Twentyman PR. Preclinical pharmacokinetics of benznidazole. British journal of cancer. 1984;50(3):291-303.

4. Raaflaub J, Ziegler WH. Single-dose pharmacokinetics of the trypanosomicide benznidazole in man. Arzneimittel-Forschung. 1979;29(10):1611-4.

5. Bosch-Nicolau P, Fernández ML, Sulleiro E, Villar JC, Perez-Molina JA, Correa-Oliveira R, et al. Efficacy of three benznidazole dosing strategies for adults living with chronic Chagas disease (MULTIBENZ): an international, randomised, double-blind, phase 2b trial. The Lancet Infectious diseases. 2024;24(4):386-94.
