# Supplementary material for: Pharmacokinetic-pharmacodynamic modeling of benznidazole and its antitrypanosomal activity in a murine model of chronic Chagas disease
Source: PLoS Negl Trop Dis. 2025 May 13;19(5):e0012968. doi: 10.1371/journal.pntd.0012968 (PMC12074391; doi:10.1371/journal.pntd.0012968)
Supplement: S1 Fig — (DOCX) [file pntd.0012968.s005.docx]

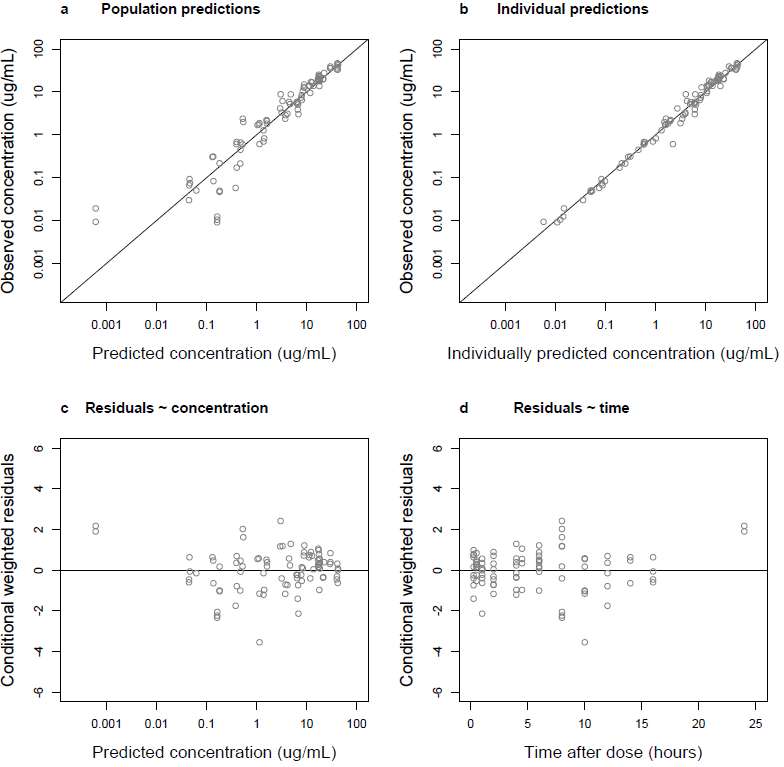


**S1 Fig.** Goodness-of-fit for the final population pharmacokinetic model of benznidazole in BALB/c mice. **A:** observed versus population predicted concentrations. **B:** observed versus individually predicted concentrations. **C:** conditionally weighted residuals versus population predicted concentrations. **D:** conditional weighted residuals versus time after dose. Observations are represented as grey circles, while solid grey lines represent the line of identity or zero line.
