# Supplementary material for: Pharmacokinetic-pharmacodynamic modeling of benznidazole and its antitrypanosomal activity in a murine model of chronic Chagas disease
Source: PLoS Negl Trop Dis. 2025 May 13;19(5):e0012968. doi: 10.1371/journal.pntd.0012968 (PMC12074391; doi:10.1371/journal.pntd.0012968)
Supplement: S2 Fig — (DOCX) [file pntd.0012968.s006.docx]

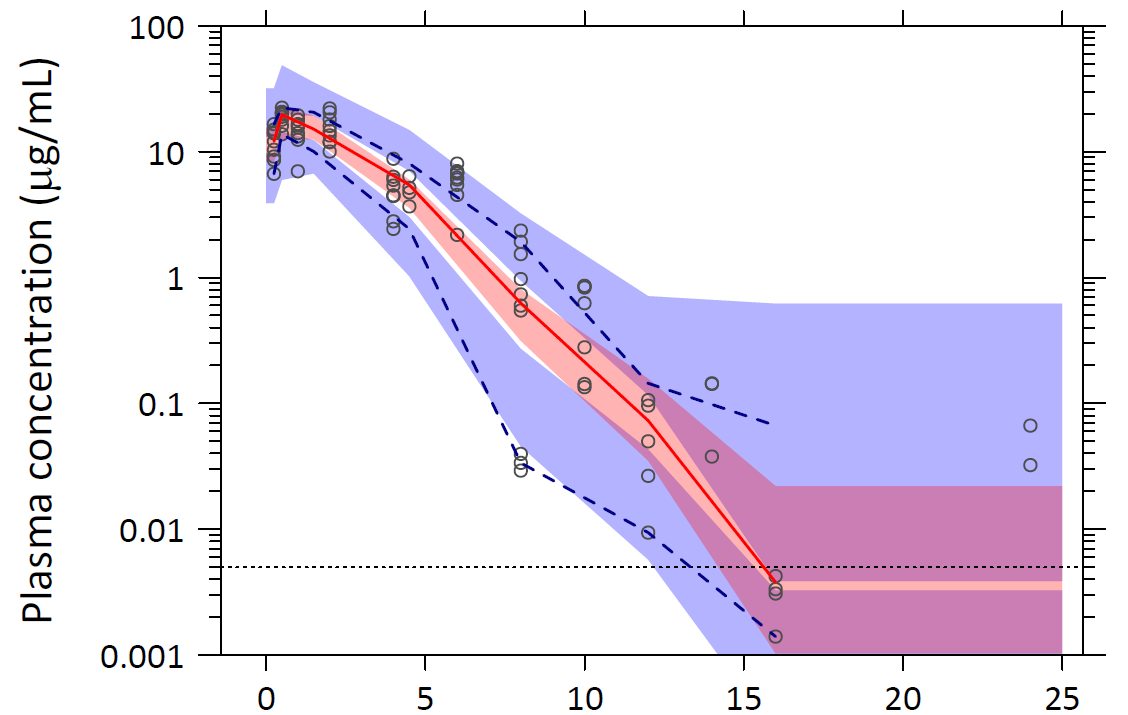


**S2 Fig.** Prediction-corrected visual predictive check of the final population pharmacokinetic model for benznidazole. Open circles represent observed plasma benznidazole concentrations from 52 female BALB/c mice across all dose groups (10, 30, and 100 mg/kg), including 90 plasma concentrations above the lower limit of quantification (LLOQ). The solid red line represents the 50th percentile (median) and the dashed blue lines represent the 5th and 95th percentiles of the observed data. The shaded areas represent the 95% confidence intervals around the simulated 5th, 50th, and 95th percentiles. The horizontal dashed line represents the LLOQ (5 ng/mL = 0.005 µg/mL)
