# Supplementary figures and images for: Pharmacokinetic-pharmacodynamic modeling of benznidazole and its antitrypanosomal activity in a murine model of chronic Chagas disease
Source: PLoS Negl Trop Dis. 2025 May 13;19(5):e0012968. doi: 10.1371/journal.pntd.0012968 (PMC12074391; doi:10.1371/journal.pntd.0012968)

**
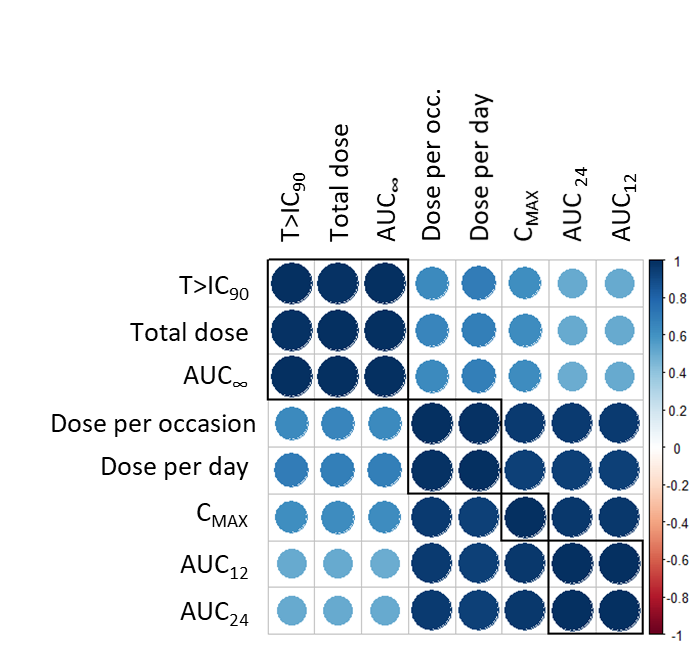
**

**S3 Fig.** Graphical representation of the correlation matrix.

Supplement: S3 Fig — (DOCX) [file pntd.0012968.s007.docx]
