# Supplementary material for: Pharmacokinetic-pharmacodynamic modeling of benznidazole and its antitrypanosomal activity in a murine model of chronic Chagas disease
Source: PLoS Negl Trop Dis. 2025 May 13;19(5):e0012968. doi: 10.1371/journal.pntd.0012968 (PMC12074391; doi:10.1371/journal.pntd.0012968)
Supplement: S1 Code — (DOCX) [file pntd.0012968.s008.docx]

**S1 Code**

NONMEM code of the final model

;; Modeler: Frauke Assmus, Ayorinde Adehin, Richard Hoglund, Joel Tarning

;; Project name: Benznidazole PK in mice

;;-------------------------------------------------------------------------------------------------------------;;

$SUBROUTINE ADVAN5 TRANS1

;;-------------------------------------------------------------------------------------------------------------;;

$MODEL

COMP=(1) ; Absorption compartment

COMP=(2) ; Central CMP

$PK

; Dose effect

COV1 = (DOSE_PER_KG/30)**THETA(5)

;;-------------------------------------------------------------------------------------------------------------;;

; Disposition parameters

TVCL = THETA(1)*((WT/0.0194)**0.75) ; Elimination clearance

CL = TVCL*EXP(ETA(1))

TVV2 = THETA(2)*((WT/0.0194)**1.00) ; Central volume

V2 = TVV2*EXP(ETA(2))

TVKA = THETA(3)*COV1 ; Absorption rate constant

KA = TVKA*EXP(ETA(3) )

TVF1 = THETA(4) ; Relative bioavailability

F1 = TVF1*EXP(ETA(4))

K20 = CL/V2

K12 = KA

S2 = V2

;;-------------------------------------------------------------------------------------------------------------;;

$ERROR

CP = A(2)/S2 ; Predicted plasma concentration

IPRED = CP

IF(IPRED.GT.0) IPRED = LOG(IPRED)

Y=IPRED+EPS(1)

W=SQRT(SIGMA(1,1))

IRES = DV-IPRED

IWRES = IRES/W

IF(AMT.GT.0) DTIM=TIME

TAD=TIME-DTIM

;;-------------------------------------------------------------------------------------------------------------;;

$THETA

(0,10.6) ; 1 CL

(0,19.7) ; 2 V2

(0, 2.18) ; 3 KA

1 FIX ; 4 F1

(-0.775) ; 5 DOSE_KA

;;-------------------------------------------------------------------------------------------------------------;;

$OMEGA

0.0298 ; IIV_CL

0.0271 ; IIV_V2

0 FIX ; IIV_KA

0 FIX ; IIV_F1

;;-------------------------------------------------------------------------------------------------------------;;

$SIGMA

0.122 ; residual error

;;-------------------------------------------------------------------------------------------------------------;;

$ESTIMATION
