# Supplementary material for: Pharmacokinetic-pharmacodynamic modeling of benznidazole and its antitrypanosomal activity in a murine model of chronic Chagas disease
Source: PLoS Negl Trop Dis. 2025 May 13;19(5):e0012968. doi: 10.1371/journal.pntd.0012968 (PMC12074391; doi:10.1371/journal.pntd.0012968)
Supplement: S1 Table — (DOCX) [file pntd.0012968.s009.docx]

**S1 Table.** UPLC-MS/MS method.

|  | **Mice plasma** |
| --- | --- |
| **Extraction** | Protein precipitation for plasma using acetonitrile (2-fold volume ratio) |
| **Calibration range** | 5 – 50.000 ng/mL |
| **Instrument** | Waters Micromass Quattro Premier coupled to a Waters Acquity UPLC |
| **Column** | Supelco Ascentis Express RP Amide column (50x2.1 mm, 2.7 μm) |
| **Mobile phase** | Acetonitrile-water gradient with 0.05% formic acid |
| **LC conditions** | Gradient cycle time: 4 min; Injection vol: 3 μL; Flow rate: 0.4 mL/min |
| **Detection** | Positive electrospray ionisation multiple-reaction monitoring mode |
| **Inter-batch precision (% RSD)** | < 10% |
| **Inter-batch accuracy (% bias)** | < 5% |
