# Supplementary material for: Pharmacokinetic-pharmacodynamic modeling of benznidazole and its antitrypanosomal activity in a murine model of chronic Chagas disease
Source: PLoS Negl Trop Dis. 2025 May 13;19(5):e0012968. doi: 10.1371/journal.pntd.0012968 (PMC12074391; doi:10.1371/journal.pntd.0012968)
Supplement: S2 Table — (DOCX) [file pntd.0012968.s010.docx]

**S2 Table.** Secondary pharmacokinetic parameter estimates, based on the final population pharmacokinetic model for benznidazole.

| **Parameter** | **10 mg** | **30 mg** | **100 mg** | **Pooled** |
| --- | --- | --- | --- | --- |
| **n mice** | 16 | 18 | 18 | 52 |
| **T_MAX_ (h)** | 0.49 (0.42 – 0.52) | 0.86 (0.77 – 0.97) | 1.47 (1.29 – 1.61) | 0.86 (0.44 – 1.58) |
| **C_MAX_ (µg/mL)** | 8.0 (7.2 – 8.4) | 19.0 (18.2 – 19.7) | 44.7 (41.8 – 46.8) | 19.0 (7.4 – 46.5) |
| **AUC_∞_ (μg×h/mL)** | 18.1 (13.7 – 19.7) | 57.3 (49.1 – 70.1) | 186 (166 – 205) | 57.3 (15.1 – 200) |
| **Terminal half life (h)** | 1.23 (0.78 – 1.43) | 1.33 (1.01 – 1.80) | 1.39 (1.05 – 1.66) | 1.33 (0.85 – 1.71) |

All values are given as median (5^th^ to 95^th^ percentile).

Abbreviations: T_MAX_, time after dose to reach the maximum concentration in plasma (C_MAX_); AUC_∞_, cumulative AUC extrapolated to infinity.
