# Supplementary material for: Pharmacokinetic-pharmacodynamic modeling of benznidazole and its antitrypanosomal activity in a murine model of chronic Chagas disease
Source: PLoS Negl Trop Dis. 2025 May 13;19(5):e0012968. doi: 10.1371/journal.pntd.0012968 (PMC12074391; doi:10.1371/journal.pntd.0012968)
Supplement: S3 Table — (DOCX) [file pntd.0012968.s011.docx]

**S3 Table.** Classification performance for logistic regression models, based on default and optimized (in blue) probability thresholds.

| **Parameter** | **Cumulative dose**  **(mg/kg)** | | **C_MAX_**  **(µg/mL)** | | **AUC_0-12_**  **(µg×h/mL)** | | **AUC_0-24_**  **(µg×h/mL)** | | **AUC_∞_**  **(µg×h/mL)** | | **T>IC_90_**  **(days)** | |
| --- | --- | --- | --- | --- | --- | --- | --- | --- | --- | --- | --- | --- |
| **Classification threshold** | Default:  50 % | Optimal:  44.4 % | Default:  50 % / | Optimal:  57.5 % | Default:  50 % / | Optimal:  55.8% | Default:  50% / | Optimal:  54.1% | Default:  50% / | Optimal:  44.0% | Default:  50% / | Optimal:  56.3% |
| **Accuracy** | 0.84 | 0.90 | 0.82 / | 0.84 | 0.82 | 0.82 | 0.82 | 0.82 | 0.84 | 0.90 | 0.90 | 0.90 |
| **Sensitivity** | 0.88 | 0.99 | 0.99 / | 0.88 | 0.99 | 0.99 | 0.99 | 0.99 | 0.88 | 0.99 | 0.99 | 0.99 |
| **Specificity** | 0.74 | 0.69 | 0.43 / | 0.74 | 0.43 | 0.43 | 0.43 | 0.43 | 0.74 | 0.69 | 0.69 | 0.69 |
| **F1 score** | 0.88 | 0.93 | 0.89 / | 0.88 | 0.89 | 0.89 | 0.89 | 0.89 | 0.88 | 0.93 | 0.93 | 0.93 |
| **Matthews Correlation Coefficient** | 0.617 | 0.753 | 0.556 | 0.556 | 0.556 | 0.556 | 0.556 | 0.556 | 0.617 | 0.753 | 0.753 | 0.753 |
| **ROC (%), (95% CI)** | 85.8 (77.2 – 94.3) | | 87.0 (80.3 – 93.7) | | 79.7 (71.3 – 88.0) | | 79.9 (71.6 – 88.2) | | 86.4 (78.0 – 94.7) | | 85.3 (76.8 – 93.8) | |

Abbreviations: C_MAX_, maximum plasma concentrations; AUC_,_ area under the concentration-time curve for up to 12 h and 24 h after dosing (AUC_12_, AUC_24_), and cumulative AUC extrapolated to infinity (AUC_∞_). T>IC_90_, Time above IC_90_ in plasma; ROC, area under the Receiver Operating Characteristics (ROC) curve.
