# Supplementary material for: Pharmacokinetic-pharmacodynamic modeling of benznidazole and its antitrypanosomal activity in a murine model of chronic Chagas disease
Source: PLoS Negl Trop Dis. 2025 May 13;19(5):e0012968. doi: 10.1371/journal.pntd.0012968 (PMC12074391; doi:10.1371/journal.pntd.0012968)
Supplement: S4 Table — (DOCX) [file pntd.0012968.s012.docx]

**S4 Table.** Sensitivity analysis: T>IC_90_ for dosing regimens in benznidazole efficacy studies, based on a range of IC_90_ values.

|  | **T>IC_90_ (days)** | | | | |
| --- | --- | --- | --- | --- | --- |
| **Dosing regimen** | **10 fold lower IC_90_** | **2 fold lower IC_90_** | **Reference (in vitro)**  **IC_90_ = 6.427 µg/mL** | **2 fold higher IC_90_** | **10 fold higher IC_90_** |
| **a)** 100 mg/kg, 10 days, QD | 4.85 | 3.49 | 2.86 | 2.19 | 0.00^a^ |
| **b)**  100 mg/kg, 5 days, QD | 2.43 | 1.75 | 1.44 | 1.11 | 0.00 |
| **c)** 50 mg/kg, 10 days, BID | 7.83 | 5.15 | 3.95 | 2.64 | 0.00 |
| **d)** 50 mg/kg, 10 days, QD | 3.90 | 2.56 | 1.96 | 1.32 | 0.00 |
| **e)** 30 mg/kg, 20 days, QD | 6.73 | 4.06 | 2.85 | 1.51 | 0.00 |
| **f)** 30 mg/kg, 10 days, QD | 3.36 | 2.01 | 1.41 | 0.75 | 0.00 |
| **g)** 30 mg/kg, 5 days, QD | 1.69 | 1.02 | 0.72 | 0.38 | 0.00 |
| **h)** 20 mg/kg, 10 days, QD | 2.96 | 1.61 | 1.01 | 0.25 | 0.00 |
| **i)** 10 mg/kg, 20 days, QD | 4.67 | 1.94 | 0.68 | 0.00 | 0.00 |
| **j)** 10 mg/kg, 10 days, QD | 2.32 | 0.97 | 0.33 | 0.00 | 0.00 |

Abbreviations: QD, once daily; BID, twice daily; T>IC_90_, Time above IC_90_ in plasma.

^a^ A value of 0.00 indicates that the plasma concentration did not reach the target concentration when assuming a 10-fold higher IC90 value in vivo compared to in vitro. This was observed for all investigated dosing regimens.
