# Supplementary material for: Pharmacokinetic-pharmacodynamic modeling of benznidazole and its antitrypanosomal activity in a murine model of chronic Chagas disease
Source: PLoS Negl Trop Dis. 2025 May 13;19(5):e0012968. doi: 10.1371/journal.pntd.0012968 (PMC12074391; doi:10.1371/journal.pntd.0012968)
Supplement: S5 Table — (DOCX) [file pntd.0012968.s013.docx]

**S5 Table.** Sensitivity analysis: univariate logistic regression diagnostics, based on a range of IC_90_ values.

| **Parameter** | **T> IC_90_ (days)** | | | | **AUC_∞_**  **(µg×h/mL)** |
| --- | --- | --- | --- | --- | --- |
|  | **10 fold lower IC_90_** | **2 fold lower IC_90_** | **Reference**  **IC_90_ = 6.427 µg/mL** | **2 fold higher IC_90_** |  |
| ***Parameter estimates*** |  |  |  |  |  |
| **Intercept (SE)** | -0.926+ | -1.680** | -1.859*** (0.521) | 1.815*** | -2.170*** (0.566) |
| **LogOdds (SE)** | 0.425*** | 0.995*** | 1.497*** (0.289) | 2.472*** | 0.0033*** (0.0006) |
| **Odds^a^** | 53.0 | 170.4 | 346.8 | 1085.1 | 0.33 |
| ***Goodness of fit*** |  |  |  |  |  |
| **AIC** | 134.3 | 119.9 | 108.5 | 96.9 | 98.8 |
| **BIC** | 139.8 | 125.5 | 114.0 | 102.5 | 104.4 |
| **Log Likelihood** | -65.126 | -57.971 | -52.240 | -46.462 | -47.412 |
| **Mc Fadden R^2^** | 0.092 | 0.192 | 0.272 | 0.352 | 0.339 |
| ***Classification performance*** | |  |  |  |  |
| **ROC (%), (95% CI)** | 74.0 (63.5 – 84.4) | 78.1 (67.7 – 88.5) | 85.3 (76.8 – 93.8) | 85.2 (76.7 – 93.7) | 86.4 (78.0 – 94.7) |

Abbreviations: SE, standard error; AIC, Aikaike information criterion; BIC, Bayesian information criterion; ; T>IC_90_, Time above IC_90_ in plasma;

*** p value < 0.001, ** p value <  0.01, + p value < 0.1

^a^Increase in odds of parasitological cure per unit increase in AUC (μg×h/mL), and Time above IC_90_ (days).
