# Supplementary material for: Pharmacokinetic-pharmacodynamic modeling of benznidazole and its antitrypanosomal activity in a murine model of chronic Chagas disease
Source: PLoS Negl Trop Dis. 2025 May 13;19(5):e0012968. doi: 10.1371/journal.pntd.0012968 (PMC12074391; doi:10.1371/journal.pntd.0012968)
Supplement: Attachment — Submitted filename: Response_to_reviewers.pdf [file pntd.0012968.s015.pdf]

Response to Reviewers

Responses to reviewer comments are highlighted in blue.

Reviewer #1

Methods

Yes, the design and methods are appropriate. I have one (probably minor) concern: The BLI model is done with the CL Brener strain, but the IC90 for their PK analysis was done using the Tulahuen strain. I wonder why they don't use the IC90 from the CL Brener strain?

The PK analysis was based on a Tulahuen strain in vitro IC90 value (17.4 µM), determined in a previously published high-content, image-based analysis (S1 text). This strain was selected because comprehensive in vitro data, including protein binding data in assay medium, were readily available. Importantly, the IC90 value for CL Brener amastigotes is similar (13.3 µM).

A systematic comparison of T. cruzi susceptibility across strains, including CL Brener and Tulahuen, conducted by Moraes et al. (see reference below), supports this finding. The study reported relatively small differences in EC50 values for benznidazole (~2.5-fold) between the two strains and consistently observed over 90% maximum efficacy across all tested strains (representing all 6 DTU units). Moraes et al. also highlighted the importance of assay methodology and controlled experimental conditions when comparing strain-specific drug susceptibility.

To account for potential variability in IC90 values, we conducted a sensitivity analysis.

For greater transparency, we have revised the Discussion to address these points:

"The IC_{90} used in our analysis was derived from the Tulahuen strain, as comprehensive in vitro data, including protein binding to assay medium, were readily available at the time of the analysis. The IC_{90} for CL Brener amastigotes in assay medium (13.3 µM) is similar to the IC_{90} for the Tulahuen strain (17.4 µM), and relatively small differences in strain susceptibility for benznidazole have been found previously [references]. To address uncertainty, we conducted a sensitivity analysis spanning a 10-fold range of IC_{90} values, demonstrating that $T>IC_{90}$ is highly sensitive to IC_{90} variability. This finding underscores the limitations of $T>IC_{90}$ as a PK/PD index parameter and the importance for careful interpretation of PK/PD targets."

References:
Moraes et al. Nitroheterocyclic compounds are more efficacious than CYP51 inhibitors against Trypanosoma cruzi: implications for Chagas disease drug discovery and development. Scientific reports. 2014;4:4703.

MacLean LM, Thomas J, Lewis MD, Cotillo I, Gray DW, De Rycker M. Development of Trypanosoma cruzi in vitro assays to identify compounds suitable for progression in Chagas' disease drug discovery. PLoS neglected tropical diseases. 2018;12(7):e0006612.

Results

Results are clearly presented.

Conclusions

The conclusions are supported by the data.

Editorial and Data Presentation Modifications?

No comments

Summary and General Comments

The manuscript by Assmus et al describes a PK-PD analysis of the antiparasitic drug, benznidazole, in the murine model of chronic T. cruzi infection. This work is justified since benznidazole remains the primary therapeutic for treating T. cruzi infection in humans. Despite it's long use since the 1970s, there remain many questions about the optimal dosing and duration of therapy to benefit chronically infected patients. In fact there are ongoing clinical trials to test shorter courses and different doses mainly to improve the poor side-effect profile while maintaining comparable efficacy. The research in this paper can theoretically help guide the clinical use of benznidazole to reproduce the PK-PD profile that is effective in mice.

For these studies, the investigators relied on data generated with the bioluminescence model of T. cruzi infection. This is the most robust animal model for determining parasitological cure of the animals. The results of using benznidazole treatment in the chronic infection model had been generated previously. The PK data with benznidazole was obtained with varying dosing regimens in uninfected mice of the same strain. Their PK results were pretty much consistent with published data. The analysis of the exposure-response relationship showed that AUC, T>IC90, and Cmax ALL demonstrated strong associations with the probability of parasitological cure when benznidazole was administered for at least five days. The high collinearity among those parameters prevented the identification of a distinct PK DRIVER of benznidazole's antitrypanosomal activity. As pointed out by the authors, detailed dose fractionation studies may be needed to better answer the fundamental question about the PK driver for this drug. They propose doing that work in future studies.

Although the studies don't lead to decisive conclusion about the exposure-response relationship between benznidazole and antiparasitic activity in chronic murine T. cruzi infection, the data do expand our general understanding of this relationship and make clear there's not a real simple answer. The authors have drawn reasonable conclusions based on the presented data. This research is a helpful addition to the Chagas disease literature. My only comment for attention was listed above about the Methods.

Thank you for your positive review and thoughtful comments.

Reviewer #2

Methods

How is chronic infection defined in this mouse model?

We appreciate your thoughtful comments and suggestions.

In this model (T. cruzi CL Brener: BALB/c mice) infections are highly reproducible. They peak in the acute stage after 14 days, and then transition to the chronic stage between days 50-70 post-infection. This is an immune-mediated process. From this point, the chronic infection, as inferred from the BLI signal, remains stable for periods beyond one year (average flux 1 x 106 p/s). As examples, see references below:

- Lewis et al. (2014) Bioluminescence imaging of chronic Trypanosoma cruzi infections reveals tissue-specific parasite dynamics and heart disease in the absence of locally persistent infection. Cell Microbiol 16, 1285-1300.
- Francisco et al. (2016) Nitroheterocyclic drugs cure experimental Trypanosoma cruzi infections more effectively in the chronic stage than in the acute stage. Sci Rep 6, 35351.

Added to the Methods section / Benznidazole efficacy in mice:

"In this model, chronic infection is defined as the stable phase following the acute stage, typically transitioning between days 50–70 post-infection in an immune-mediated process. Beyond this point, the chronic infection remains stable for over a year, as inferred from consistent BLI flux levels (average ~1×10^6 p/s).[references]"

What was the rationale for the (varying) sample sizes for each treatment dose / duration? Animal numbers per group seem very different from one another (e.g. how were sample sizes defined / calculated?)

For this work, we used BLI data derived from a number of drug discovery projects within our group. Animal numbers, together with treatment dose and duration, reflected the requirements and design of the individual experiments. This approach allowed us to capture a wide range of conditions, as well as optimizing animal usage in accordance with our welfare guidelines.

Some regimens (e.g., 100 mg/kg for 10 days) served as references in multiple experiments for benchmarking efficacy and represented a mix of historical and newly generated data. Other regimens were included to explore less commonly evaluated treatment scenarios and investigate dose-response relationships. An advantage of using population-based PK/PD modelling is that it allows for pooling of unbalanced data from different sources and experiments with different design.

This has been clarified in the revised Methods section / Benznidazole efficacy in mice:

"The sample sizes for each regimen were based on data from multiple drug discovery projects, reflecting the specific requirements and design of the individual experiments. For instance, the 100 mg/kg for 10 days regimen served as a reference across multiple experiments, while other regimens were included to assess new dosing scenarios and explore dose-response relationships"

"The final population PK model for benznidazole in satellite mice…" What are "satellite mice"?

Satellite mice refer to non-infected animals specifically used for pharmacokinetic studies to characterize the pharmacokinetic behaviour of benznidazole. These mice were distinct from those used in efficacy studies, allowing for richer PK sampling.

To clarify this, the following text has been added:

- **Methods / PK data**: "Satellite mice refer to non-infected animals specifically used in pharmacokinetic studies to provide rich sampling and characterize benznidazole's pharmacokinetic behavior."
- **Methods / Simulations**: "The final population PK model for benznidazole in non-infected satellite mice was utilized to simulate plasma concentration-time profiles for the various dosing regimens investigated in benznidazole efficacy studies."

I understand that there were 92 plasma samples included in the PK analysis? From 118 mice? Is that correct? Were there multiple samples from some mice and none from others? Maybe, also add this information to Fig 1 too (i.e. total number of benznidazole measurements in the model)

We clarified the number of plasma samples and mice in the PK analysis, updating the Methods, Results, and Fig. 1 caption:

- **Methods / Population PK analysis (clarified details and moved up for emphasis):** "A total of 110 post-dose PK samples from satellite PK studies in uninfected BALB/c mice were collected across three benznidazole dose groups (10, 30, and 100 mg/kg). Two outlying PK samples were identified and excluded from all analysis. The benznidazole PK data for the 10

mg/kg and 30 mg/kg dose groups at 8 h and 12 h after dose, respectively, were censored as all plasma levels beyond these time points were below the LLOQ. For the 100 mg/kg dose group, benznidazole levels were included for the entire PK sampling time frame (up to 24 h). After censoring, 93 PK samples remained in the dataset, of which 90 were above the LLOQ and were included in the final population PK analysis."

- **Results / PK (summary added):** "The pharmacokinetic properties of benznidazole were characterized using PK data from 52 female, non-infected BALB/c mice. Each mouse received a single oral dose of 10 mg/kg (n = 16), 30 mg/kg (n = 18), or 100 mg/kg (n = 18) benznidazole. Sparse PK sampling was employed, with 1 to 3 blood samples collected per mouse. In total, 90 plasma concentrations above the LLOQ were included in the final population PK analysis."
- **Fig. 1 caption (details added):** "Open circles represent observed plasma benznidazole concentrations from 52 female BALB/c mice across all dose groups (10, 30, and 100 mg/kg), including 90 plasma concentrations above the lower limit of quantification (LLOQ)."

Results

“parasitological cure was achieved in over 90% of chronically infected mice when treated once daily with 100 mg/kg benznidazole for 5 or 10 days (scenario a and b), or twice daily with 50 mg/kg benznidazole for 10 days (scenario c). In contrast, once daily dosing with 50 mg/kg benznidazole for 10 days (scenario d) cured only 66.7% of infected mice”. A measure of uncertainty of these estimates should be included (e.g. 95% CI). The way this is written, it seems to imply that 50 mg/kg BID x 10 days was clearly different from 50 mg/kg OD x 10 days, whereas the first group only had 3 mice in it. At least add the N of each group in the text (so that the reader doesn’t have to go back and forth to the table all the time to understand the text).

We have clarified the sample sizes for each dosing regimen in the Results section to ensure transparency, reducing the need for readers to refer back to the table.

Updated results/Benznidazole efficacy:

"In brief, parasitological cure was achieved in over 90% of chronically infected mice when treated once daily with 100 mg/kg benznidazole for 5 days (17/17 mice) or 10 days (25/27 mice; scenarios a and b). Similarly, twice daily dosing with 50 mg/kg per administration (100 mg/kg/day) for 10 days cured all mice (6/6; scenario c). In contrast, once daily dosing with 50 mg/kg for 10 days cured only 66.7% of infected mice (2/3; scenario d). Pronounced variability in drug efficacy was also observed with 30 mg/kg benznidazole: cure rates reached approximately 80% in mice treated for 10 days (9/10; scenario f) or 20 days (23/29 mice; scenarios e), while a shorter treatment duration of 5 days failed to cure any mice (0/9; scenario g). Similar non-curative outcomes were observed for lower benznidazole doses (10 and 20 mg/kg, scenarios h-j). With these dosing schedules, a reduction in parasite burden was achieved, but none (0/6; 10 days) or only 16.7% of mice (1/6; 20 days) were ultimately cured."

Table 1 (and every time that the dosing schedules are mentioned): When (e.g.)50 mg/kg BID is mentioned, do the authors mean that each time (in this case twice per day) the mice received a 50 mg/kg dose (i.e. a 100 mg/kg/day dose), or a 50 mg/kg /day dose in 2 administrations (i.e. 25 mg/kg twice a day)?

In this study, the dosing schedules represent the dose administered per administration. 50 mg/kg BID indicates that each administration delivered 50 mg/kg, resulting in a total daily dose of 100 mg/kg.

This has been clarified in Table 1 and throughout the manuscript:

- **Table 1 (footnote added):** [a]Dosing schedules indicate the dose per administration. For BID regimens, the total daily dose is the sum of the doses administered twice daily (e.g., 50 mg/kg BID = 100 mg/kg/day)."
- **Results / Benznidazole efficacy:** "Similarly, twice daily dosing with 50 mg/kg per administration (100 mg/kg/day) for 10 days cured all mice (6/6; scenario c)."

“In the present study, a higher dose was associated with a significantly slower absorption rate (ΔOFV = -31.3), as indicated by a decrease in KA (power function). At the lowest, medium and highest dose, KA was estimated as 5.11, 2.18 and 0.86 h-1, respectively.” Please add some estimate of uncertainty around the point estimates.

The uncertainty for the KA estimate (2.18 h^{-1}) at the median dose is provided in Table 2, including its 95% confidence interval (1.65–3.03) and relative standard error (15.9%).

Also, were the higher doses administered in higher liquid volumes?

The Methods section has been revised to include the formulation concentrations used for each dosing regimen, along with additional details on the preparation and administration process.

Updated Methods/PK data:

"A fixed dose volume of 0.2 mL per mouse was used for all dosing regimens. Benznidazole formulations were prepared to achieve concentrations of approximately 1 mg/mL, 3 mg/mL, and 10 mg/mL for the 10, 30, and 100 mg/kg dose levels, respectively. The exact dose administered to each mouse was calculated based on the measured concentration of the formulation and the animal's body weight. Formulations contained 5% (v/v) DMSO and a suspension vehicle composed of 0.5% (w/v) hydroxypropyl methylcellulose, 0.5% (v/v) benzyl alcohol and 0.4% (v/v) Tween 80 in Milli-Q water. The formulations were vortexed, sonicated and dosed to mice within 1.5 hours of preparation. Before each individual administration, formulations were actively resuspended to ensure uniform dispersal of benznidazole in the vehicle."

” Goodness-of-fit diagnostics (AIC, BIC, Mc Fadden R2) indicated slightly stronger explanatory power for AUC∞ in binary regression compared to CMAX”. How precise is the estimation of CMAX in a model with such sparse sampling?

The estimation of C_{MAX} in our study is based on population PK modeling, which accounts for sparse sampling designs by pooling data across all mice to estimate typical parameter values and variability. Visual predictive checks (VPCs) suggest that the model adequately captures the observed plasma concentration-time profiles. However, we acknowledge the inherent limitations of sparse sampling in precisely estimating individual C_{MAX} values, as well as the limitation of using median concentrations rather than individual concentrations for PK/PD modeling.

This limitation has already been addressed in the Discussion section under PK properties (see response to Reviewer #, No. 2).

Also, wouldn’t cumulative dose be a better explanatory variable? Was this tested?

Multivariate analysis: similar to above, was cumulative dose included in the analysis?

We agree that cumulative dose is a relevant metric and tested it in both univariate and multivariate regression models. However, in this dataset, cumulative dose and simulated median AUC∞ values showed a perfect correlation ($R^2 = 1$, see Table 3). Consequently, the logistic regression model yielded identical performance metrics for both parameters, as shown in Table 4 and Supplementary Table S4.

While cumulative dose provides valuable information, we focused on plasma exposure parameters like AUC∞ due to their greater translational value. AUC∞ directly reflects plasma exposure, which can be adjusted for protein binding and potentially serve as a reference for human dose predictions. However, it is important to note that the primary driver of efficacy remains unclear.

To address the reviewer's suggestion, we have included predictions based on cumulative dose in Table 5 to provide a more comprehensive overview, while maintaining our emphasis on plasma exposure metrics.

This has been clarified in the Results section / Univariate logistic regression (added):

"Given the high correlation between cumulative dose and AUC∞ in this dataset, the performance of the logistic regression model was identical for both metrics. However, we focus on plasma exposure metrics (such as AUC_{∞} or C_{MAX}) due to their greater potential translational value for human dose predictions."

Conclusions

"Administering benznidazole as a suspension at doses of 30 mg/kg and 100 mg/kg resulted in delayed absorption (TMAX = 0.86 h and 1.47 h, respectively) compared to the clear 10 mg/kg solution (TMAX = 0.49 h)." As suggested above, some detail on actual administrated liquid volumes would be useful here

Please see our response above (Updated Methods/PK data), where this information has been clarified:

"A fixed dose volume of 0.2 mL per mouse was used for all dosing regimens. […]."

- "we also noted a similar trend towards lower clearance at higher doses. While flip flop kinetics or saturable elimination cannot be ruled out" What about enterohepatic circulation?

No evidence of a double-peak profile, characteristic of enterohepatic recirculation, was observed in the plasma concentration-time data for benznidazole in mice in this study. While the possibility of enterohepatic circulation cannot be completely excluded, the data available do not support incorporating this mechanism into the pharmacokinetic model.

Editorial and Data Presentation Modifications?

The title could be a bit more clear on what the authors mean as pharmacodynamics in their experimental setting.

It is important to define what "PD" means. In this case, it seems that the authors refer to drug response (defined as absence of bioluminescence after immunosuppression with cyclophosphamide, I guess).

Maybe the title should add information on this (e.g. "Pharmacokinetic-pharmacodynamic analysis of T Cruzi elimination after benznidazole treatment in a murine model of chronic Chagas disease"

The title has been updated to better reflect the pharmacodynamic aspect as antitrypanosomal activity:

Pharmacokinetic-Pharmacodynamic Modeling of Benznidazole and Its Antitrypanosomal Activity in a Murine Model of Chronic Chagas disease

It may also be worth considering (and clarifying) that this is not really a murine model of chronic chagas disease (e.g. there's no real chronic cardiac or Gi pathology similar to what humans develop, etc). This is a murine model of chronic T cruzi infection.

The host:parasite combination used in this study is a murine model of chronic Chagas disease. While human disease comprises a wide spectrum of tissue pathology and clinical symptoms that develop over years, *T. cruzi*-infected mice also display key pathological features that are hallmarks of chagasic heart disease. Notably, mice develop chronic myocarditis and cardiac fibrosis as early as 84 days post-infection, in a manner that shares similarities with human pathology (references below).

Importantly, if mice are cured with benznidazole during the acute stage of infection, they do not develop heart pathology. However, when treatment is postponed until the chronic stage (>100 days), it is too late to prevent the accumulation of cardiac damage. If extendable to humans, this implies that widespread chemotherapeutic intervention targeted at early-stage infections should play a crucial role in reducing Chagas disease morbidity at a population level.

References:

Francisco et al. (2018) Assessing the effectiveness of curative benznidazole treatment in preventing chronic cardiac pathology in experimental models of Chagas disease. Antimicrob. Agents Chemother. 62, e00832-18.

Lewis MD, Fortes Francisco A, Taylor MC, Burrell-Saward H, McLatchie AP, Miles MA, et al. Bioluminescence imaging of chronic Trypanosoma cruzi infections reveals tissue-specific parasite dynamics and heart disease in the absence of locally persistent infection. Cellular microbiology. 2014;16(9):1285-300.

Summary and General Comments

This is an interesting animal PK-PD model. It adds some insight to the literature available, and attempts to link the observations to human observations (e.g. in S4) at least to a limited extent. Some of the commnents made in S4 could be in the actual discussion to help readers link the mouse model to what actually goes on in humans.

While the manuscript is already quite detailed, we agree that adding a brief discussion linking the mouse model to human observations would provide some insights into translational challenges.

Updated Discussion / PK/PD relationship:

"An exploratory analysis comparing simulated plasma concentration - time profiles of benznidazole in mice and humans is shown in the Supplementary Information (S4, Text). The analysis suggests that the human standard dosing regimen achieves higher total plasma exposure and a longer duration above the target concentration than the efficacious mouse regimen, but substantially lower peak concentrations. These findings highlight a potential disconnect between exposure levels in mice and humans. However, this remains speculative, as neither the key driver of efficacy nor the optimal human dosing regimen has been established."

Reviewer #3

Methods, Results, Conclusions, Editorial & Data Presentation Modifications?

please refer to the general comments

Summary and General Comments

The study by Assmus et al. investigated the quantitative PK/PD relationship of benznidazole in a murine model of chronic Chagas disease using the population PK approach. This represents the first of its kind using efficacy data generated from the BLI imaging data. The described work is highly relevant to the scope of PLOS-NTD and advances our understanding of factors driving benznidazole efficacy in

chronic Chagas diseases. In general, the manuscript has been clearly written and organized with sufficient details on the methodologies employed and the results presented. Discussion was found to be pertinent and intriguing, and conclusions were supported by the study results. However, several moderate/minor concerns should be addressed prior to acceptance.

1. Authors should use the opportunity to assess factors that both drive efficacy and could potentially be employed for future therapeutic drug monitoring (TDM) purposes. Currently, Cmax, AUC and T>IC90 have been identified as the driving factors, however these measurements will be impossible for use in clinics to monitor treatment and predict efficacy. More realistic ones are C_trough values (e.g., at 12h, 24h or before last dose). It'd be very interesting to know whether these factors drive efficacy as well.

Thank you for your insightful comments and valuable suggestions.

We agree that exploring clinically relevant pharmacokinetic parameters, such as C_{trough}, could be valuable for developing TDM strategies. However, in this preclinical model, C_{trough} has limited relevance as a predictive factor due to benznidazole's short half-life in mice.

Simulations demonstrated that C_{trough} values approached zero before the next dose in both once-daily and twice-daily regimens (Fig. 2). While AUC, Cmax, and T>IC_{90} are less straightforward to measure for clinical TDM, they showed stronger associations with antitrypanosomal activity in *T. cruzi*-infected mice. Further research is needed to determine whether C_{trough} becomes more relevant in humans, where slower clearance may enhance its predictive utility.

We have added relevant text to the manuscript:

- **Results/Simulated BZN exposure:** "Trough concentrations (C_{trough}) approached zero before the next dose for both once-daily and twice-daily regimens and were therefore not considered further."
- **Discussion/PK/PD relationship:** "Interestingly, simulations revealed that C_{trough} approached zero before the next dose in both once-daily and twice-daily regimens, reflecting the short half-life of benznidazole in mice. This points to a limited relevance of C_{trough} as a predictor of efficacy in this preclinical model. Alternative PK metrics may provide valuable insights for therapeutic drug monitoring in humans, where benznidazole exhibits a longer half-life, but this remains speculative and requires further investigation in clinical studies."

2. Authors should also acknowledge the limitation of using satellite PK from healthy mice rather than PK/exposure from the infected mice used in the efficacy study. It was mentioned (Line 526) that PK from uninfected and infected mice were similar, which is important. But this does not take away the additional insights one can get from assessing exposure and efficacy on the same subject, due to interindividual and intraindividual variability and ability to identify factors that drive efficacy on the individual level and can be potentially used for TDM.

The logistics of the experimental design limited our ability to assess exposure and efficacy in the same animals. However, as the reviewer notes, the similarity of PK between uninfected and infected mice is a key factor that supports the validity of our approach.

We have revised the Discussion section / PK properties to acknowledge this limitation:

"However, relying on PK data from healthy satellite mice instead of directly measuring plasma exposure in the infected mice used for efficacy studies limits the ability to fully capture inter- and intra-individual variability. Simultaneous assessment of exposure and efficacy in the same infected mice could provide valuable insights into factors influencing treatment outcomes."

3. Line 153, please provide the dose formulation concentrations (e.g., mg/ml) used for different dose regimens, as it was reasoned later (Line 500) that differences in the dose suspension concentration may have led to difference in absorption kinetics.

The Methods section has been revised to include the formulation concentrations used for each dosing regimen, along with additional details on the preparation and administration process.

Updated Methods/PK data:

"A fixed dose volume of 0.2 mL per mouse was used for all dosing regimens. Benznidazole formulations were prepared to achieve concentrations of approximately 1 mg/mL, 3 mg/mL, and 10 mg/mL for the 10, 30, and 100 mg/kg dose levels, respectively. The exact dose administered to each mouse was calculated based on the measured concentration of the formulation and the animal's body weight. Formulations contained 5% (v/v) DMSO and a suspension vehicle composed of 0.5% (w/v) hydroxypropyl methylcellulose, 0.5% (v/v) benzyl alcohol and 0.4% (v/v) Tween 80 in Milli-Q water. The formulations were vortexed, sonicated and dosed to mice within 1.5 hours of preparation. Before each individual administration, formulations were actively resuspended to ensure uniform dispersal of benznidazole in the vehicle."

4. Line 157, why was food kept from mice for this group?

Food and drinking water were available ad libitum at dosing and up to 6 hours post-dose for all dose groups. For the 100 mg/kg group, a preliminary experiment identified that definition of the exposure profile was confounded by an atypically high degree of inter-animal variability at sample times corresponding to the overnight (dark cycle) period. While probing the underlying basis of that variability was beyond the scope of the study, the apparent variability was reduced when access to food was withdrawn prior to the end of the light cycle (i.e. at 6 hours post-dose). However, as absorption is rapid (T_{MAX} < 2 hours), this was unlikely to affect absorption, and food effects were not incorporated into the population PK model.

Updated Methods section / PK data:

"Food and drinking water were available ad libitum at dosing and up to 6 hours post-dose for all dose groups."

5. Line 198, add refs for these allometric scaling factors.

Reference has been added in the Methods section / PK analysis:

Gabrielsson, J., D. Weiner, and J. Gabrielsson, Pharmacokinetic & pharmacodynamic data analysis : concepts and applications. 4th ed. rev. and expanded. ed. Pharmacokinetic and pharmacodynamic data analysis. 2006, Stockholm: Apotekarsocieteten.

6. Remove the strikethrough on line 181 in SI.

The strikethrough on line 181 in the SI has been removed.

7. Fig 1: it’s unclear what type of observed data are plotted here. Are they from the same dose regimen group? Which one?

Fig. 1 is a visual predictive check (VPC) showing all observed data from the single-dose pharmacokinetic study. The observed data include plasma concentrations from all dose groups (10, 30, and 100 mg/kg). The Methods section specifies the dose groups and sampling strategy.

The figure caption has been updated for clarity:

"Open circles represent observed plasma benznidazole concentrations from 52 female BALB/c mice across all dose groups (10, 30, and 100 mg/kg), including 90 plasma concentrations above the lower limit of quantification (LLOQ)."
