# Supplementary material for: Pharmacokinetic-pharmacodynamic modeling of benznidazole and its antitrypanosomal activity in a murine model of chronic Chagas disease
Source: PLoS Negl Trop Dis. 2025 May 13;19(5):e0012968. doi: 10.1371/journal.pntd.0012968 (PMC12074391; doi:10.1371/journal.pntd.0012968)
Supplement: Attachment — Submitted filename: Response to Reviewers 3.pdf [file pntd.0012968.s017.pdf]

Review Rebuttal

Review Comment

I appreciate the addition of Fig S2 for transparency. Although upon closer examination, most of observed data (open circles) in Fig 1 and Fig S2 do not match each other. Please have them checked and corrected. In addition, please prepare and include a data table in the supplemental information to show all the time and plasma concentration values for the observed data.

Response

We thank the Reviewer for the comment and careful review. Indeed, the plotted observations in Figures 1 and S2 are different, but this is expected. We wish to clarify and reiterate that Figure S2 shows a **prediction-corrected** visual predictive check (pcVPC), in which both the predictions and observations are adjusted from their original values. The transformation in a pcVPC is computed according to the equation[1] below:

$$\ln(pcY_{ij}) = \ln(Y_{ij}) + [\ln(PRED_{bin}) - \ln(PRED_{ij})]$$

Here,

- Y_{ij} is the observation or prediction for the ith individual and j^{th} time-point,
- pcY_{ij} is the prediction-corrected observation/prediction,
- $PRED_{ij}$ is the typical population prediction for the ith individual and j^{th} time-point, and
- $PRED_{bin}$ is the median of the typical population predictions for the specific bin of independent variables.

This means that both the observations and predictions are corrected based on the model prediction. These transformations explain the expected differences between the open circles presented in Figures 1 and S2. This methodology is commonly used in pharmacometric modelling and was developed in order to pool data and provide a diagnostic tool to simultaneously evaluate data that are expected to be different, such as dose groups and different patient populations. However, since the data are corrected, these diagnostic tools often produce pharmacokinetic curves that appear somewhat non-intuitive and can be challenging for non-pharmacometricians to interpret. This is why we opted to add this in the supplement, as it might be of interest for a specific group of readers but not for the larger audience of PLOS NTD. We hope that this explanation solves any residual concerns, and we encourage the reviewer to request the individual level data from the MORU data access committee for secondary analyses.

Reference

1. Bergstrand M, Hooker AC, Wallin JE, Karlsson MO. Prediction-corrected visual predictive checks for diagnosing nonlinear mixed-effects models. *AAPS J* **13**, 143-151 (2011).
