# Supplementary material for: Pharmacokinetic-pharmacodynamic modeling of benznidazole and its antitrypanosomal activity in a murine model of chronic Chagas disease
Source: PLoS Negl Trop Dis. 2025 May 13;19(5):e0012968. doi: 10.1371/journal.pntd.0012968 (PMC12074391; doi:10.1371/journal.pntd.0012968)
Supplement: Attachment — Submitted filename: Response_to_reviewers.docx [file pntd.0012968.s016.docx]

**Response to Reviewers**

Responses to reviewer comments are highlighted in blue.

***Methods***

- Reviewer #1: This is a revised manuscript. The authors have adequately addressed the concerns raised in my initial review (Reviewer 1).
- Reviewer #2: the authors have addressed all my questions
- Reviewer #3: (No Response)

***Results***.

- Reviewer #1: This is a revised manuscript. The authors have adequately addressed the concerns raised in my initial review (Reviewer 1).
- Reviewer #2: the authors have addressed all my questions
- Reviewer #3: (No Response)

***Conclusions***

- Reviewer #1: This is a revised manuscript. The authors have adequately addressed the concerns raised in my initial review (Reviewer 1).
- Reviewer #2: the authors have addressed all my questions
- Reviewer #3: (No Response)

***Editorial and Data Presentation Modifications?***

- Reviewer #1: Accept
- Reviewer #2: the authors have addressed all my questions
- Reviewer #3: (No Response)

***Summary and General Comments***

- Reviewer #1: This is a revised manuscript. The authors have adequately addressed the concerns raised in my initial review (Reviewer 1).
- Reviewer #2: the authors have addressed all my questions

Thank you very much for your positive reviews and valuable feedback.

- Reviewer #3: Authors addressed all my previous concerns, except for the confusion on Figure 1. The way the Fig. 1 is currently presented does not serve the purpose of visual predictive check of the pop-pk model. What’s the point of averaging benznidazole plasma concentrations from all three dose groups (10, 30 and 100 mpk), i.e., the solid red line? Plasma concentrations are supposed to be different from different doses. The shaded areas do not make sense either. What dose was used in the model-simulation to generate these? A reasonable way is to delineate the data among different dose groups using different symbols, plot median and 5th and 95th percentiles of the data for each dose group, and simulate 95% CI with shaded areas for specific dose. This way will allow visual check of overlap between the data and simulation.

Thank you for your thoughtful feedback. We appreciate your concerns regarding the interpretation of Figure 1. However, we respectfully disagree with the suggestion to stratify the VPC by dose group.

The primary reason for presenting a pooled VPC rather than a dose-stratified graph is data sparsity. Given the limited number of animals per dose group, stratification would not provide reliable diagnostics. Furthermore, due to non-random censoring, the dataset does not fully support the use of a prediction-corrected VPC for robust model evaluation, as indicated in the literature (Bergstrand et al., 2009). Therefore, we have opted to present the non-prediction-corrected VPC. That said, we acknowledge the importance of transparency and have now included the prediction-corrected VPC in the Supplementary Information (Fig. S2) for completeness. To clarify this decision, we have revised the Results section- PK of benznidazole as follows:

“It is important to note that non-random censoring of data below the LLOQ can complicate the interpretation of prediction-corrected VPCs [32]. In our study, PK data were censored at 8 hours (10 mg/kg) and 12 hours (30 mg/kg) post-dose, while no censoring was applied in the 100 mg/kg dose group. Therefore, the non-prediction-corrected VPC is presented (Fig. 1). For completeness, the prediction-corrected VPC is provided in the Supplementary Information (Fig. S2).”

Reference: Bergstrand M, Hooker AC, Karlsson MO, editors. Visual Predictive Checks for Censored and Categorical data. PAGE; 2009; St.Petersburg, Russia.
